# BH3-Mimetics- and Cisplatin-Induced Cell Death Proceeds through Different Pathways Depending on the Availability of Death-Related Cellular Components

**DOI:** 10.1371/journal.pone.0056881

**Published:** 2013-02-21

**Authors:** Vicente Andreu-Fernández, Ainhoa Genovés, Angel Messeguer, Mar Orzáez, Mónica Sancho, Enrique Pérez-Payá

**Affiliations:** 1 Laboratory of Peptide and Protein Chemistry, Centro de Investigación Príncipe Felipe, Valencia, Spain; 2 Department of Chemical and Biomolecular Nanotechnology, Instituto Química Avanzada de Cataluña (CSIC), Barcelona, Spain; 3 Instituto de Biomedicina de Valencia, IBV-CSIC, Valencia, Spain; The University of Texas MD Anderson Cancer Center, United States of America

## Abstract

**Background:**

Owing to their important function in regulating cell death, pharmacological inhibition of Bcl-2 proteins by dubbed BH3-mimetics is a promising strategy for apoptosis induction or sensitization to chemotherapy. However, the role of Apaf-1, the main protein constituent of the apoptosome, in the process has yet not been analyzed. Furthermore as new chemotherapeutics develop, the possible chemotherapy-induced toxicity to rapidly dividing normal cells, especially sensitive differentiated cells, has to be considered. Such undesirable effects would probably be ameliorated by selectively and locally inhibiting apoptosis in defined sensitive cells.

**Methodology and Principal Findings:**

Mouse embryonic fibroblasts (MEFS) from Apaf-1 knock out mouse (MEFS KO Apaf-1) and Bax/Bak double KO (MEFS KO Bax/Bak), MEFS from wild-type mouse (MEFS wt) and human cervix adenocarcinoma (HeLa) cells were used to comparatively investigate the signaling cell death-induced pathways of BH3-mimetics, like ABT737 and GX15-070, with DNA damage-inducing agent cisplatin (cis-diammineplatinum(II) dichloride, CDDP). The study was performed in the absence or presence of apoptosis inhibitors namely, caspase inhibitors or apoptosome inhibitors. BH3-mimetic ABT737 required of Apaf-1 to exert its apoptosis-inducing effect. In contrast, BH3-mimetic GX15-070 and DNA damage-inducing CDDP induced cell death in the absence of both Bax/Bak and Apaf-1. GX15-070 induced autophagy-based cell death in all the cell lines analyzed. MEFS wt cells were protected from the cytotoxic effects of ABT737 and CDDP by chemical inhibition of the apoptosome through QM31, but not by using general caspase inhibitors.

**Conclusions:**

BH3-mimetic ABT737 not only requires Bax/Bak to exert its apoptosis-inducing effect, but also Apaf-1, while GX15-070 and CDDP induce different modalities of cell death in the absence of Bax/Bak or Apaf-1. Inclusion of specific Apaf-1 inhibitors in topical and well-localized administrations, but not in systemic ones, to avoid interferences with chemotherapeutics would be of interest to prevent chemotherapeutic-induced unwanted cell death which could improve cancer patient care.

## Introduction

Current anti-tumour treatments based in inducing apoptosis target cancer cells and rapidly dividing normal cells as well as other especially sensitive differentiated cells. Therefore, these treatments do not differentiate between malignant and normal cells. Chemotherapy causes toxicity, leading to side effects like those reported for apoptosis-inducing and DNA-damaging agent cisplatin (cis-diammineplatinum(II) dichloride, CDDP), which induces ototoxicity [1] and alopecia [2]. These undesirable effects may be ameliorated by the discovery of new more specific cell death-inducing drugs [3], or by selectively and locally inhibiting apoptosis in defined sensitive cells.

The discovery of the components of the apoptosis signaling pathway is providing the basis for novel targeted therapies that can induce death in cancer cells. Then BCL-2 antagonists as the chemotherapeutical drugs called BH3-mimetics are in clinical phase II [4]. On the other hand, apoptosis inhibitors-based drugs may have the potential to locally attenuate chemotherapy-induced side effects if the effective dose of apoptosis inducer (chemotherapeutic drug) *versus* apoptosis inhibitor is defined. Current synthetic apoptosis inhibitors include caspase inhibitors [5] and apoptosome inhibitors [6].

The proposal of developing BH3-mimetics as chemotherapeutic drugs originates from understanding the role of the Bcl-2 protein family in regulating the intrinsic apoptotic pathway by controlling mitochondria outer membrane permeability (MOMP). The anti-apoptotic members of this family (Bcl-2, Bcl-xL, Bcl-W, Mcl-1 and A1) are characterized by the homology of four regions denominated Bcl-2 homology domains (BH1, BH2, BH3 and BH4), pro-apoptotic members, Bax, Bak and Bok, which share domains BH1-3, while the BH3-only proteins (e.g., Bad, Bid, Bim, Noxa and Puma) contain only the BH3 region [7]. BH3-only proteins promote apoptosis by suppressing anti-apoptotic proteins at the mitochondria and the endoplasmic reticulum or by directly activating Bax and Bak [8]. The anti- and pro-apoptotic balance of Bcl-2 proteins is deregulated in cancer cells [9]. Extensive work was performed to elucidate the process whereby protein-protein interactions between Bcl-2 protein family members commit cells to apoptosis. As a unified model, and under homeostatic conditions, anti-apoptotic Bcl-2 family members present a hydrophobic groove that interacts with the BH3 domain of pro-apoptotic effectors (Bax and Bak) or the BH3-only proteins to allow their sequestration, as well as the inhibition of MOMP. Apoptotic stimuli release Bax and Bak from the hydrophobic groove to induce oligomerization at the mitochondria membrane and MOMP. Therefore, cytochrome *c* (Cyt *c*) and Smac/Diablo proteins are released from the mitochondrial intermembrane space [10]. Cyt *c* binds to apoptosis protease-activating factor-1 (Apaf-1) to induce apoptosome assembling that recruits and activates initiator caspase-9, which further activates effector caspases, inducing apoptotic cell death [11].

The small molecule compounds developed as inhibitors of anti-apoptotic Bcl-2 proteins, generically named BH3-mimetics such as ABT737 (Abbott Laboratories) or obatoclax (GX15-070, Gemin X Biotechnologies), release pro-apoptotic binding partners and suffice to induce apoptosis. ABT737 binds selectivity to anti-apoptotic Bcl-2, but has a low affinity to Mcl-1 and A1 [12], [13]. GX15-070 has been proposed to influence the activity of the Bak/Mcl-1 and Bim/Mcl-1 complexes [14] to induce mitochondrial-mediated apoptosis, which would imply Bax/Bak-mediated MOMP and apoptosome-mediated activation of caspases. However, in some cell lines that are relevant for disease, GX15-070-treatment has also been described to render phenotypic cell characteristics which could be associated with GX15-070 activities, including autophagy, independently of mitochondrial-mediated apoptosis. The cytotoxic activity of GX15-070 and ABT737 in Bax/Bak double knockout cells has also been reported [15], [16], while the role of the apoptosome is unclear as it is still to be explored in detail. This is particularly relevant for studying the activity of BH3-mimetics in cells with low Apaf-1 contents that correlate with resistance to chemotherapeutic treatments [17], [18] and for preclinically evaluating a new class of apoptosis inhibitors targeting the apoptosome [19], [20], which are currently being evaluated as agents to locally prevent chemotherapy-induced secondary effects. It would then be of interest to comparatively analyze the activity of BH3-mimetics and CDDP (as a representative of established cytotoxic drugs) in cells in which Apaf-1 has been genetically deleted and to also analyze whether apoptosome inhibitors can inhibit BH3-mimetics-induced cell death.

Here we analyzed the ability of BH3-mimetics GX15-070 and ABT737 to induce cell death in mouse embryonic fibroblasts (MEFS) from Apaf-1 knockout (KO) mouse (MEFS KO Apaf-1) and in MEFS from wild-type mouse (MEFS wt) in the presence and absence of the apoptosis inhibitors Z-Val-Ala-Asp(OMe)-fluoromethylketone (zVADfmk – a general caspase inhibitor [5]) and QM31, an apoptosome inhibitor [19], [20]. The results were comparatively evaluated with the effects of CDDP under the same experimental conditions and were extended to MEFS from Bax/Bak double KO mouse (MEFS KO Bax/Bak) and to human cervix adenocarcinoma (HeLa) cells.

## Materials and Methods

### Cell culture, treatments and chemicals

ABT737 and GX15-070 were from Abbott Laboratories and from SelleckBio, respectively; cis-diammineplatinum(II) dichloride (cisplatin, CDDP), rapamycin and 3-methyladenine (3MA) were obtained from Sigma Aldrich. QM31 is a perhydro-1,4-diazepine-2,5-dione whose general synthetic method has been recently reported [21]. The HeLa cell line was purchased from ATCC, and MEFS [22], [23] were provided by Dr. Guido Kroemer (MEFS wt and KO Bax/Bak) and Dr. Francesco Cecconi (MEFS wt and KO Apaf-1). All the cell lines were grown in Dulbecco's Modified Eagle's Medium (DMEM) supplemented with 10% fetal bovine serum (FBS). Cultures were maintained at 37°C in a 5% CO_2_ atmosphere. Cell media and FBS were purchased from GIBCO BRL Life Technologies. When indicated, cells were treated with 1 µM of GX50-070, 25 µM of ABT737, 30 µM of rapamycin and 30 µM of CDDP. When required, 10 mM MA, 10 µM QM31 or 5 µM zVAD were administered 30 min after treatment addition, and cells were maintained in culture for 24 h. Assays were carried out between passage 6 and 10, in all cases.

### Determination of caspase activity

All cell extracts were prepared from 1.5×10^5^ cells seeded in 6-well plates. After 24 h, cells were treated as indicated above and were then scrapped and washed with PBS. Pellets were resuspended in extraction buffer (50 mM PIPES, 50 mM KCl, 5 mM EDTA, 2 mM MgCl2, 2 mM DTT) supplemented with protease inhibitor cocktail (Sigma) and kept on ice for 5 min. Once pellets were frozen and thawed three times, cell lysates were centrifuged at 14000 rpm for 5 min and supernatants were collected. Quantification of the total protein concentration was performed using the BCA protein assay (Thermo Scientific). Total protein (50 µg) was mixed with 200 µL of caspase assay buffer (PBS, 10% glycerol, 0.1 mM EDTA, 2 mM DTT) containing 20 µM of the Ac-DEVD-afc (Enzo Life Sciences) caspase-3 specific substrate. Caspase activity was continuously monitored following the release of fluorescent afc at 37°C using a Wallac 1420 Workstation (λexc = 400 nm; λem = 508 nm). Caspase-3 activity was expressed as the increase of relative fluorescence units per min (A.U.).

### Flow cytometry

After drug treatment, the cell culture medium was collected to retain floating cells and attached cells were dislodged using 0.5% Trypsin-EDTA (GIBCO). Floating and attached cells were combined and harvested by centrifugation. The cell pellets were suspended in 100 µl binding buffer (10 mM HEPES pH 7.4, 140 mM NaCl, 2.5 mM CaCl_2_) and incubated with 10 µl FITC Annexin V (BD Biosciences) and 10 µl of DRAQ7 (6 µM; Biostatus) for 10 min at 37°C. Staining for Annexin V and DRAQ7 was assessed by flow cytometry on a FC500 instrument (Beckman Coulter) followed by data analysis using FlowJo software (Tree Star Inc).

### MTT mitochondrial dysfunction assay

Mitochondrial functionality was measured by a 3-(4,5-dimethylthiazol-2-yl)-2,5-diphenyltetrazolium bromide (MTT) colorimetric assay. Cells were cultured in sterile 96-well microtiter plates at a seeding density of 1500 cells/well for the MEFS lines and 2000 cells/well for HeLa cells. After seeding, cells were left to adhere to the plate overnight, and then they were treated with the compounds of interest and incubated at 37°C for 24 h. MTT reagent (5 mg/ml in PBS) was added to each well and plates were further incubated for 4 h at 37°C. Finally, the medium was removed and the precipitated formazan crystals were dissolved in optical grade DMSO. Plates were read at 570 nm on a Wallac 1420 workstation.

### Trypan blue exclusion assay

Cells were seeded in 6-well plates at a cellular density of 1.5×10^5^ cells. After 24 h, cells were treated as described before. Cells were detached and 0.5% trypan blue dye was added in solution. Live cells possess intact cell membranes that exclude the dye, whereas dead cells do not. Unstained (viable) and stained (non-viable) cells were counted separately in a hemacytometer and the total number of viable cells in the population was calculated.

### Nuclear staining

The cells cultured on coverslips were stained with 300 nM 4′-6-diamidino-2-phenylindole (DAPI) solution. The morphology of the cells' nuclei was observed using a fluorescence microscope (Leica Vertical DM6000) at an excitation wavelength of 350 nm. Nuclei are considered to have the normal phenotype when they glow brightly and homogenously. Apoptotic nuclei can be identified by either the condensed chromatin gathering at the periphery of the nuclear membrane or a total fragmented morphology of nuclear bodies.

### Immunoblotting

Whole cell extracts were obtained by lysing cells in a buffer containing 25 mM Tris-HCl pH 7.4, 1 mM EDTA, 1 mM EGTA, 1% SDS, plus protease and phosphatase inhibitors. The protein concentration was determined by the BCA protein assay. Cell lysates were resolved by SDS-PAGE, transferred to nitrocellulose membranes, blocked with 5% non fat milk, washed with 0.1% Tween/PBS and incubated overnight with a specific primary antibody. Membranes were washed and probed with the appropriate secondary antibody conjugated to horseradish peroxidase for enhanced chemiluminescence detection (Amersham Pharmacia Biotech). The antibody against LC3 (#2775) came from Cell Signaling and α-tubulin antibody (#T8203) was from Sigma-Aldrich.

### Statistical analysis

All the values represent the mean ± s.d. of at least three independent experiments. Statistical significance was determined by one-way ANOVA using the Graph Pad software, *p*<0.05 was designated as statistically significant.

## Results and Discussion

**Figure 1:**
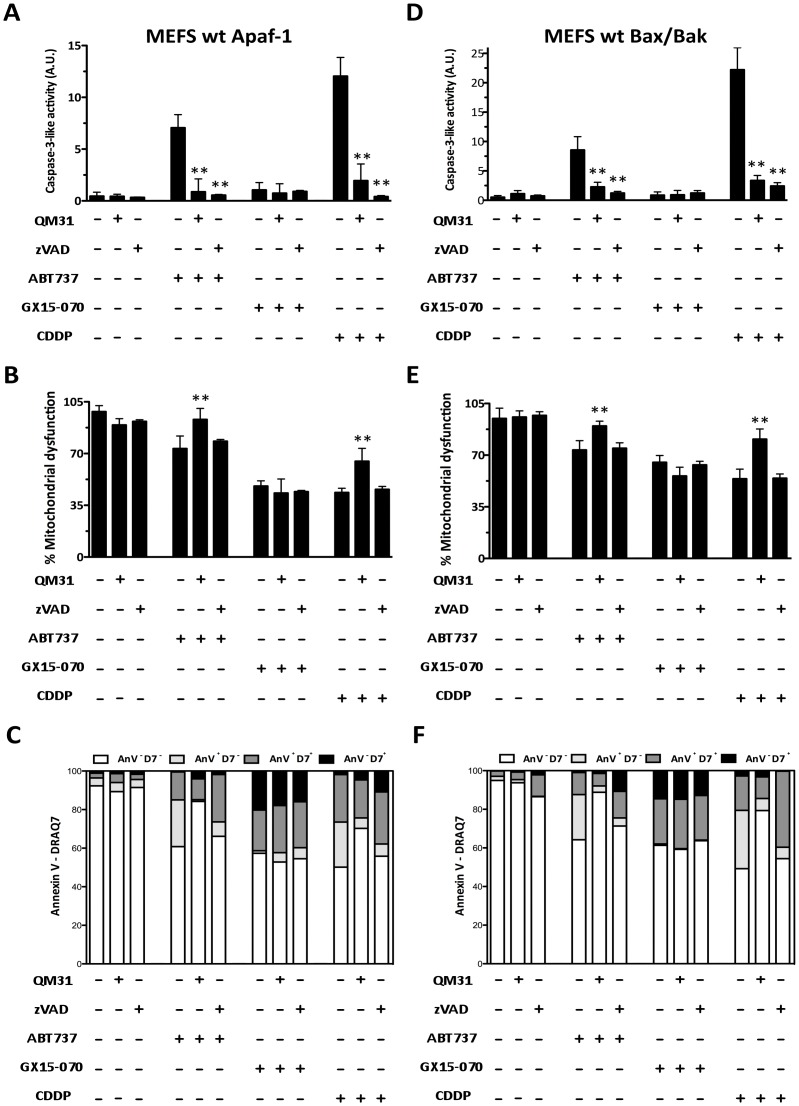
Apaf-1 inhibitor QM31 prevents cell death in non tumor cells treated with both ABT737 and CDDP, but not in cells treated with GX15-070. (**A and D**) Caspase 3-like activity was measured in MEFS wt Apaf-1 and MEFS wt Bax/Bak treated with ABT737 (25 µM), GX15-070 (1 µM) and CDDP (30 µM) in the presence or absence of QM31 (10 µM) and zVADfmk (5 µM). (**B and E**) Mitochondrial dysfunction was measured by an MTT assay under the same conditions described above. Bars represent the mean of three experiments ± s.d. (**p<0.05). (**C and F**) Apoptotic cell death was determined by flow cytometry with FITC Annexin V and DRAQ7. Data are representative results of three independent experiments.

**Figure 2:**
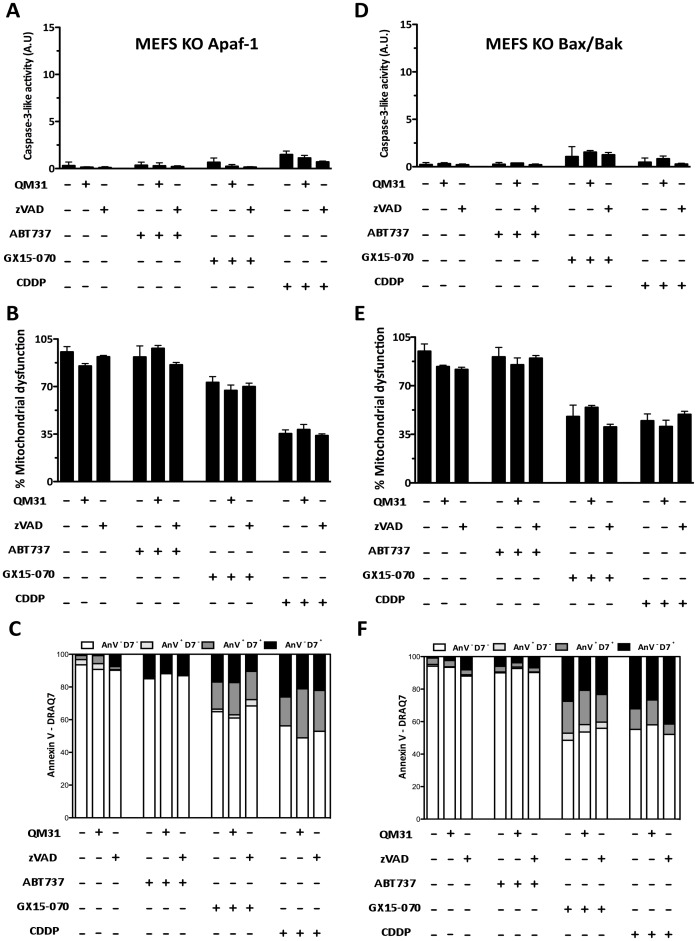
GX15-070 and CDDP induce caspase 3 independent cell death in Apaf-1 and Bax/Bak deficient cells. (**A and D**) Caspase 3-like activity was measured in MEFS KO Apaf-1 and MEFS KO Bax/Bak treated with ABT737 (25 µM), GX15-070 (1 µM) and CDDP (30 µM) in the presence or absence of QM31 (10 µM) and zVADfmk (5 µM). (**B and E**) Mitochondrial dysfunction was measured by an MTT assay under the same conditions described above. Bars represent the mean of three experiments ± s.d. (**C and F**) Apoptotic cell death was analyzed by flow cytometry with FITC Annexin V and DRAQ7. Data are representative results of three independent experiments.

**Figure 3:**
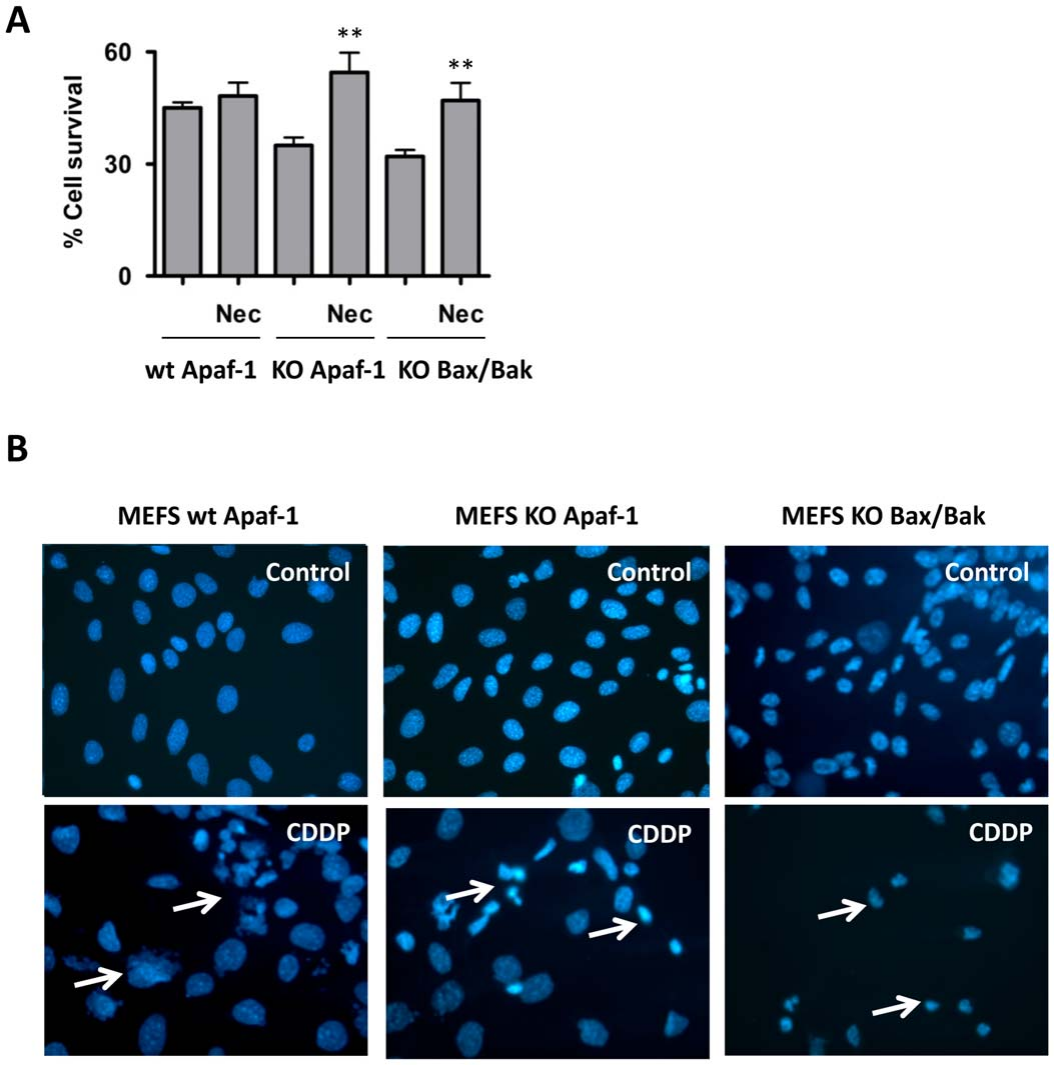
Non apoptotic cell death upon CDDP treatment in Apaf-1- and Bax/Bak-deficient cells. (**A**) Cell survival was measured by trypan blue exclusion upon treatment with CDDP (30 µM) in the presence or absence of necrostatin (Nec; 100 µM). Bars represent the mean of three experiments ± s.d. (**p<0.05). (**B**) MEFS wt Apaf-1, MEFS KO Apaf-1 and MEFS KO Bax/Bak were stained with DAPI upon CDDP (30 µM) treatment. Nuclei are considered to have the normal phenotype when glowing bright and homogenously. Apoptotic nuclei can be identified by the fragmented morphology of nuclear bodies. White arrows indicate dying cells.

Embryonic fibroblasts from wild-type mouse (MEFS wt Apaf-1 and MEFS wt Bax/Bak) [23] were treated with ABT737, GX15-070 or cisplatin (cis-diammineplatinum(II) dichloride, CDDP), either alone or in combination with apoptosome inhibitor compound QM31 [19], [20], or with broad spectrum caspase inhibitor Z-Val-Ala-Asp(OMe)-fluoromethylketone (zVADfmk). They were evaluated at 24 h post-treatment. ABT737 and CDDP treatments induced activation of caspase-3, which was inhibited by zVADfmk and by QM31. However, when cells were treated with GX15-070, only residual caspase-3 activity was observed (Fig. 1A and 1D). Cell viability was determined by MTT (Fig. 1B and 1E) to find that ABT737- and CDDP-induced death (20% and 60%, respectively) was inhibited by QM31, but not by zVADfmk, while the cell death induced by GX15-070 (around 50%) was not inhibited by either zVADfmk or QM31. Annexin V/DRAQ7 flow cytometry assays corroborate viability and apoptotic cell death results (Fig. 1C and 1F). The same experiments were conducted in Apaf-1 knockout (KO) mouse embryonic fibroblasts (MEFS KO Apaf-1) [23], in MEFS KO Bax/Bak [22] and in cervix adenocarcinoma cells (HeLa). In MEFS KO Apaf-1 (Fig. 2A) and MEFS KO Bax/Bak (Fig. 2D), none of the treatments induced caspase-3 activity, while cell viability was unaffected by the ABT737 treatment, but decreased with both GX15-070 and CDDP treatments (Fig. 2B and 2E). GX15-070- and CDDP-induced cell death in these cells was not inhibited upon apoptosome or caspase inhibition. Consequently, treatments with QM31 and zVADfmk did not significantly modify the percentage of Annexin V stained cells (Fig. 2C and 2F). These results suggest that in the absence of key death-related cellular components, such as the Bcl-2 proteins Bax and Bak and the apoptosome constituent protein Apaf-1, ABT737-triggering signaling is not fully perceived by the cell, while CDDP-depending signaling found caspase-independent cell death pathways. CDDP-induced cell death was partially recovered by necrostatin-1 (Nec), an inhibitor of RIPK1 in MEFS KO Apaf-1 and MEFS KO Bax/Bak (Fig. 3A), suggesting that necroptosis (a form of programmed necrosis that depends on activity of RIPK1) could participate in CDDP-induced death in these cells. In fact, nuclear staining upon CDDP treatment showed non apoptotic cell death in MEFS KO Apaf-1 and MEFS KO Bax/Bak cells (Fig. 3B), while treatment induced canonical apoptotic bodies in MEFS wt Apaf-1, indicating that DNA damaging agents may activate alternative cell death pathways when the intrinsic pathway of apoptosis is blocked.

**Figure 4:**
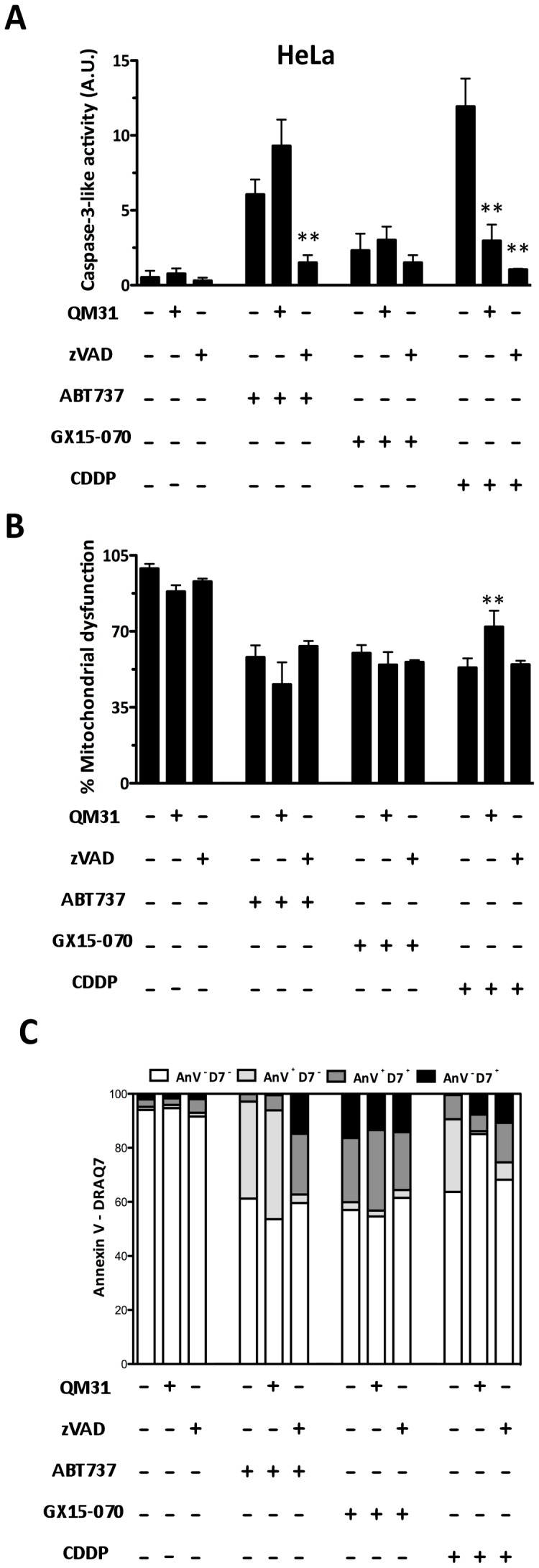
Apaf-1 inhibition does not protect tumor HeLa cells from death induced by ABT737 and GX15-070. (**A**) Caspase 3-like activity was measured in the HeLa cells treated with ABT737 (25 µM), GX15-070 (1 µM) and CDDP (30 µM) in the presence or absence of QM31 (10 µM) and zVADfmk (5 µM). (**B**) Mitochondrial dysfunction was measured by an MTT assay under the same conditions described above. Bars represent the mean of three experiments ± s.d. (*p<0.1; **p<0.05). (**C**) Apoptotic cell death was analyzed by flow cytometry with FITC Annexin V and DRAQ7. Data are representative results of three independent experiments.

In human cervix adenocarcinoma (HeLa) cells, rather than inducing caspase-3 activity, GX15-070 induced a type of cell death that was not inhibited by zVADfmk or QM31 (Figs. 4A, 4B and 4C), which correlates with the phenotypes observed in all the MEFS cell lines. CDDP induced caspase-3 activation, which was inhibited in the presence of QM31 or zVADfmk, and also cell death (Fig. 4A, 4B and 4C). CDDP-induced death was partially prevented by QM31, but not by zVADfmk (Fig. 4B and 4C). Nonetheless, the zVADfmk inhibition of ABT737-induced caspase-3 activity was unable to protect cells from dying (Fig. 4B and 4C). Interestingly, and unlike the results found in the MEFS wt, apoptosome inhibition by QM31 did not inhibit ABT737-induced caspase-3 and cell death (Fig. 4A and 4C).

**Figure 5:**
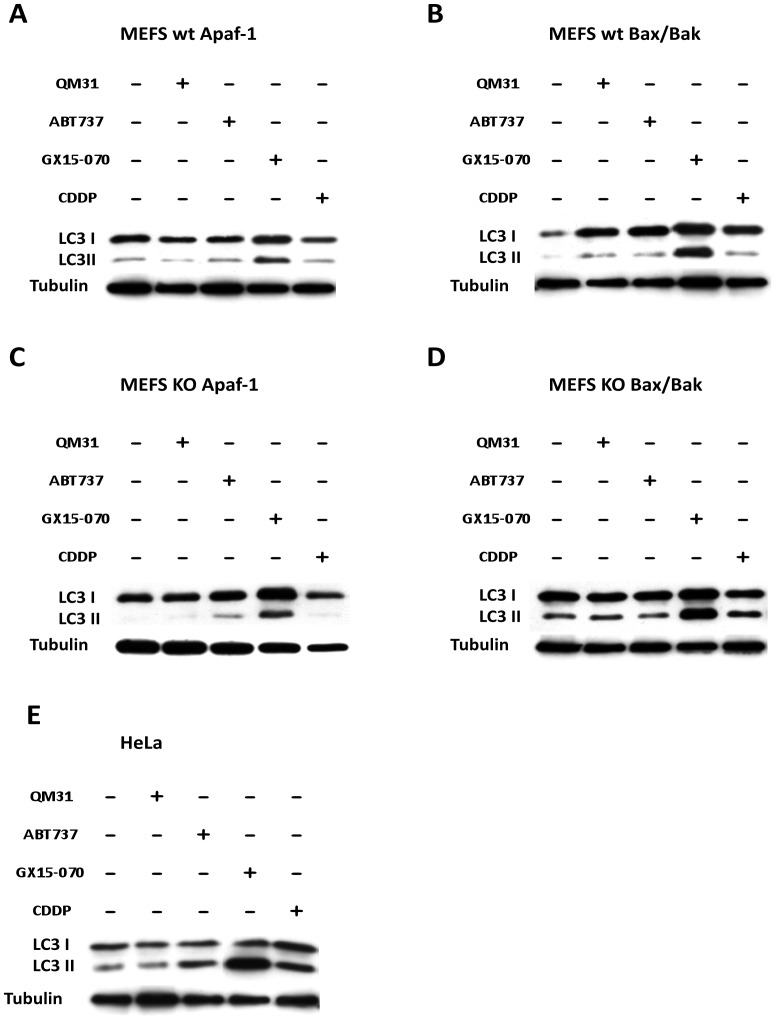
GX15-070 promotes the activation of the autophagic pathway via LC3 in all the cell lines. (**A–E**) LC3 detection in MEFS wt Apaf-1, MEFS wt Bax/Bak, MEFS KO Apaf-1, MEFS KO Bax/Bak and HeLa cells treated with ABT737 (25 µM), GX15-070 (1 µM), CDDP (30 µM ) and QM31 (10 µM).

**Figure 6:**
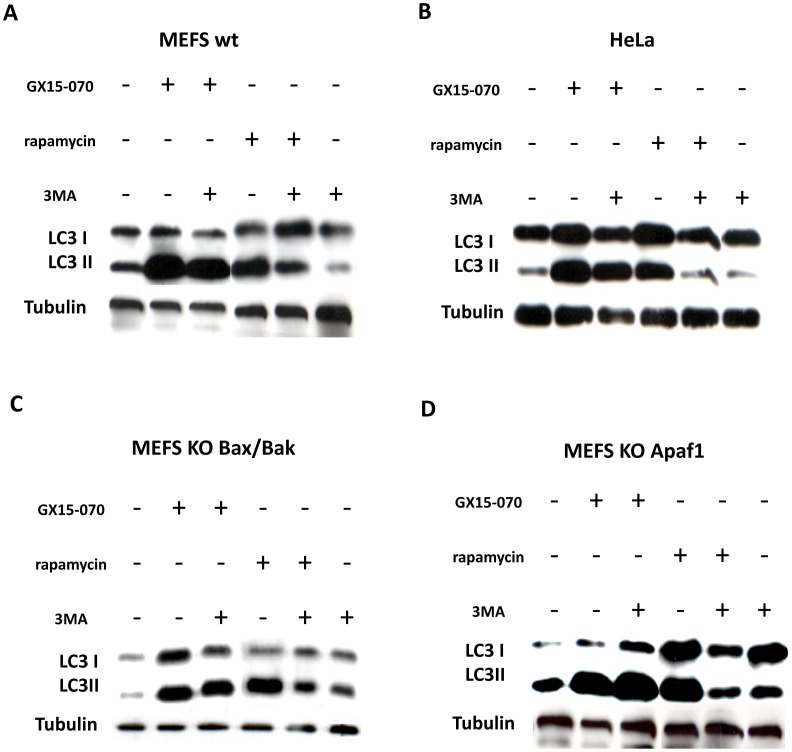
GX15-070 activates Beclin-1 non-dependent autophagy in all the cell lines. (**A–D**) LC3 immunoblotting in the MEFS wt Apaf-1, MEFS KO Apaf-1, MEFS KO Bax/Bak and HeLa cell lines treated with rapamycin (30 µM) and GX15-070 (1 µM) for 24 h in the presence or absence of 3MA (10 mM).

To proceed with an initial analysis of the cell death pathway induced by GX15-070 in the MEFS wt Apaf-1, MEFS wt Bax/Bak, MEFS KO Apaf-1, MEFS KO Bax/Bak, and HeLa cells, we analyzed the expression of anti-apoptotic proteins Bcl-2, Bcl-xL and Mcl-1 and found no significant changes (data not shown). We also explored the induction of autophagy. Autophagy is a catabolic process involving the formation of autophagosomes and autolysosomes. Light chain 3 (LC3, a mammalian ortholog of yeast Atg8 - [24]) is essential for autophagosome formation and can be used as a reporter protein. When the process of autophagy proceeds, LC3-I (the cytosolic form) it is processed to the autophagosomal membrane-bound LC3-II form [24]. The LC3-II form increased considerably with GX15-070 treatment (Fig. 5A–D), suggesting that evaluated GX15-070-induced cell death was mediated by autophagy activation in all the cell lines. The activity of III phosphoinositide 3-kinase (PI3K III) is important in Beclin-1 (the human ortholog of yeast Atg-6)-induced autophagy [25], and 3-methyladenine (3MA – an inhibitor of PI3K III) is commonly used to determine the dependence of Beclin-1 in autophagy. 3MA did not modify GX15-070-induced LC3 processing (Fig. 6). Therefore, GX15-070-induced autophagy in both MEFS wt and MEFS KO Apaf-1 is independent of Beclin-1, as also reported for MEFS KO Bax/Bak and HeLa cells [15]. As an internal control, we induced autophagy by rapamycin and found that rapamycin-induced autophagy was inhibited by 3MA in all four cell lines analyzed (Fig. 6).

**Figure 7:**
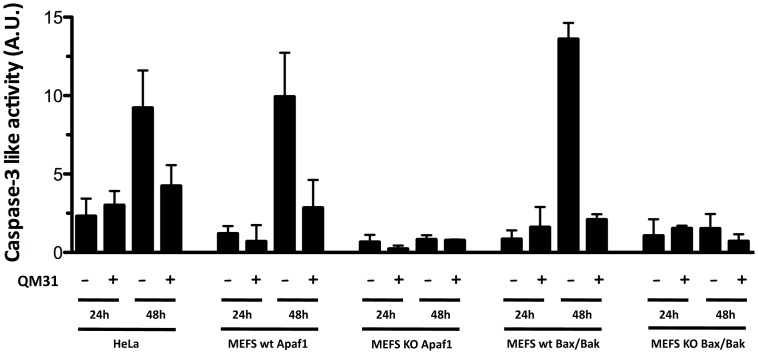
GX15-070 induces caspase 3-like activity after 48 h in HeLa and MEFs wt. Caspase 3-like activity was measured at 24 h and 48 h upon treatment with GX15-070 (1 µM) in the presence or absence of QM31 (10 µM) in HeLa, MEFS wt Apaf-1, MEFS KO Apaf-1, MEFS wt Bax/Bak and MEFS KO Bax/Bak.

It has been reported that GX15-070 is able to induce apoptosis and autophagy in several cell lines [26]. Thus we performed a time-course analysis to examine whether GX15-070-treatment induces both autophagy and apoptosis. We used LC3 conversion as a marker of autophagy (Fig. 5) and caspase-3 activity as a marker of apoptosis. After 24 h we did not observe GX15-070-induced caspase-3 activation in the cell lines analyzed in the present study (Fig. 7). At 48 h however, we noted that GX15-070-induced caspase-3 activity in both MEFS wt and in HeLa cells. GX15-070-induced apoptosis at 48 h was inhibited by apoptosome inhibitor QM31. In contrast, GX15-070 did not induce apoptosis in MEFS KO Bax/Bak and in MEFS KO Apaf-1 at 48 h. These results suggest that GX15-070 can induce multiple cell death pathways, such as caspase-dependent apoptosis and autophagy. Nevertheless, GX15-070-induced apoptosis is not only dependent in Bax/Bak, as previously demonstrated [15], [26], but also in Apaf-1.

In conclusion, the present study reveals that BH3-mimetic ABT737 not only requires Bax/Bak to exert its apoptosis-inducing effect, but also Apaf-1, indicating the exclusive targeting of ABT737 to Bcl-2 anti-apoptotic proteins. ABT737 upon binding to Bcl-2 and Bcl-xL removes the anti-apoptotic activity of these proteins in pro-apoptotic Bax/Bak and induces MOMP. However, MOMP-dependent signaling needs the components of the apoptotic pathway downstream of mitochondria, such as the formation of the apoptosome, to induce cell death. Hence, ABT737 treatments to cancer cells would have less side effects to differentiated cells containing low levels of Apaf-1, such as neurons and cardiomyocytes [27], [28], than other treatments with lesser dependence of Apaf-1. In contrast, BH3-mimetic GX15-070 and DNA damage-inducing CDDP induce cell death in the absence of both Bax/Bak and Apaf-1. While GX15-070 induces mainly autophagy-based cell death at 24 h, a cell fraction dies by apoptosis at longer times post-treatment. On the other hand, CDDP induces necroptosis when apoptosis signaling pathway is not available. Our results extend findings by describing not only the sensitivity of different cells to the cell-inducing agents explored, but also the behavior of current apoptosis inhibitors, which could be useful in topical applications aimed to diminish unwanted cell death. Non tumor cells, as demonstrated herein with MEFS wt, could be protected from the cytotoxic effects of ABT737 and CDDP by the chemical inhibition of the apoptosome through QM31, which lowered caspase-3 activity and improved cell survival, while the use of caspase inhibitors prevented caspase activation, but did not improve survival. This scenario correlates with proposals in mammals that solely caspase inhibition, downstream of MOMP delays, and in defined circumstances, modifies the outcome rather than preventing cell death [29]. However, the autophagy-based cell death induced by GX15-070 was not prevented by QM31 or caspase inhibitors. These results will be of interest when defining future combination therapies where the systemic administration of cytotoxic agents, which aims to kill malignant cells, could be locally counteracted by apoptosis inhibitors. For instance, dermatopic and intra-cochlear administration of apoptosome inhibitors would probably find applications as anti-alopecia and anti-ototoxic agents, respectively, for anti-cancer treatments based on ABT7373 and CDDP.
